# Arbutin overcomes tumor immune tolerance by inhibiting tumor programmed cell death-ligand 1 expression

**DOI:** 10.7150/ijms.92419

**Published:** 2024-11-11

**Authors:** Ching-Han Liu, Jing-Ru Weng, Li-Hsien Wu, Rui-Yang Song, Ming-Der Huang, Xin-He Wu, Chia C. Wang, Che-Hsin Lee

**Affiliations:** 1 Division of Cardiology, Department of Internal Medicine, Kaohsiung Armed Forces General Hospital, Kaohsiung, Taiwan.; 2 Department of Marine Biotechnology and Resources, National Sun Yat-sen University, Kaohsiung 80424, Taiwan.; 3 Department of Biological Sciences, National Sun Yat-sen University, Kaohsiung, Taiwan.; 4 Aerosol Science Research Center, National Sun Yat-sen University, Kaohsiung, Taiwan.; 5 Department of Chemistry, National Sun Yat-sen University, Kaohsiung 80424, Taiwan.; 6 College of semiconductor and advanced technology Research, National Sun Yat-sen University, Kaohsiung 80424, Taiwan.; 7 Department of Medical Laboratory Science and Biotechnology, Kaohsiung Medical University, Kaohsiung 80708, Taiwan.; 8 Department of Medical Research, China Medical University Hospital, China Medical University, Taichung, Taiwan.

**Keywords:** Arbutin, programmed cell death protein ligand-1, tumor immune tolerance

## Abstract

Arbutin, predominantly derived from the bearberry plant, exhibits promising immunomodulatory properties. Given its ability to influence the programmed cell death-ligand 1/ programmed cell death-1 (PD-L1/PD-1) pathway, it is emerging as a potential alternative treatment for cancer. A reduced expression of PD-L1, as seen after arbutin treatment, can bolster immune responses critical step in effective tumor immunotherapy. However, the molecular mechanism by which arbutin inhibits PD-L1 is still incompletely known. The expression of PD-L1 was decreased after tumor cells were treated with arbutin. Arbutin can downregulate the expression of PD-L1 on the cell surface via the protein kinase B (AKT)/mammalian target of rapamycin (mTOR) pathway. The findings suggest the protective role of arbutin and provide novel insights into immunotherapy, which involves inhibiting the AKT/mTOR signaling pathway. Arbutin might serve as a potential therapeutic agent alone or in combination with other treatments.

## Introduction

Arbutin is a natural hydroquinone glucoside predominantly sourced from the bearberry plant but has also been identified in at least 45 other plant families 1. Functioning as a shield against free radical-mediated and enzymatic membrane cleavage in plants, arbutin has therapeutic significance in humans 2. It is employed in cosmetics for skin whitening due to its strong tyrosinase inhibitory activity, which curbs melanin production 3. Moreover, arbutin possesses other therapeutically significant properties: anti-inflammatory, anti-oxidative, anti-microbial, and potential antitumor capabilities 4, 5. Studies have highlighted its cytotoxic nature against various tumor cell lines 6. One mechanism through which arbutin achieves its anti-tumor effects is by inducing apoptosis, as confirmed *in vitro* 7. Its impact on tumor-immune interactions remains to be fully explored.

Cancer, a genomic disorder, manifests through genomic instability and the accumulation of myriad point mutations during tumor evolution. This genomic diversity gives rise to tumor antigens, which the immune system perceives as foreign, initiating a cellular immune response 8. Effective immune responses can potentially eliminate or impede malignant cells 9. However, tumor cells have devised various strategies to circumvent immune surveillance, thereby neutralizing antitumor immune responses 10.

The past decades have witnessed significant strides in immunotherapy, a paradigm that bolsters the patient's immune system to target malignant cells 11. Among the advances, immune checkpoint inhibitors, specifically, those targeting programmed cell death-1 (PD-1), programmed cell death-ligand 1 (PD-L1), indoleamine 2,3-dioxygenase (IDO), and cytotoxic T lymphocyte antigen 4 (CTLA-4) 12-14. Their promising therapeutic efficacy has been demonstrated in various cancers, with some even gaining regulatory approval. Notably, PD-L1 inhibitors are associated with fewer immune-related side effects, positioning them as promising immunotherapeutic agents for diverse tumors 13, 14.

PD-L1, primarily expressed in activated immune cells and some epithelial cells, particularly under inflammation, plays a crucial role in immune tolerance. Its interaction with PD-1 impedes T cell functions, fostering tumor immune evasion 13. Historical studies have also pointed to tumor-associated the role of PD-L1 in promoting immune suppression 15. Beyond its immune checkpoint functions, PD-L1 also has intrinsic roles in cancer cells, such as promoting cell proliferation, survival, and metastasis. PD-L1 can activate the activated protein kinase B (AKT)/mammalian target of the rapamycin (mTOR) pathway within tumor cells, enhancing their survival and growth. PD-L1 has an intrinsic role in promoting tumor progression, independent of its well-established immunomodulatory effects. PD-L1 signaling influences various cellular processes, such as the transforming growth factor β (TGF-β) pathway and epithelial-mesenchymal transition 16, epidermal growth factor receptor (EGFR) signaling 17, and cellular metabolism 18. By influencing this pathway, PD-L1 contributes to tumor progression independently of its immune-regulatory functions. Although direct studies on arbutin's effects on PD-L1 are limited, there is a possibility that arbutin may influence cancer pathways that involve PD-L1. Arbutin has been shown to have anti-proliferative effects on certain cancer cells by inhibiting oxidative stress and related pathways 19. Given that PD-L1 can promote tumor proliferation, epithelial-mesenchymal transition (EMT), and immune evasion.

This study aims to ascertain whether arbutin can modulate immune checkpoints and diminish tumor immune tolerance by suppressing PD-L1 expression. Our results indicate that arbutin regulates PD-L1 expression in tumor cells by targeting the AKT/mTOR pathway. Furthermore, animal models confirm the potential of arbutin in alleviating tumor immune tolerance *in vivo*. In essence, arbutin might pave the way for a novel mechanism in immune checkpoint inhibition. By modulating the AKT/mTOR pathway, arbutin may promote programmed cell death in cancer cells, thereby inhibiting tumor growth.

## Materials and Methods

### Reagents, Cell Lines, Plasmids,* Salmonella* and Mice

Arbutin, cisplatin and cobalt chloride (CoCl_2_) were purchased from Sigma-Aldrich (Sigma-Aldrich, St. Louis, MO, USA). Deionized water was use to solubilize arbutin. Adherent cells murine melanoma cells (B16F10) 20 and murine lung carcinoma (LL2) 21 were cultured in Dulbecco's modified Eagle's medium (DMEM) added with 1% penicillin and 10% heat-inactivated fetal bovine serum at 37 ℃and 5% CO^2^ incubator. Suspension cells EL4 cell line (mouse T lymphocyte; (ATCC TIB-39™)) was cultured in Roswell Park Memorial Institute (RPMI) medium 1640 added with 1% penicillin, 0.05 mM 2-Mercaptoethanol and 10% heat-inactivated fetal bovine serum at 37 ℃and 5% CO^2^ incubator. The EL4 cells were derived from the C57BL/6 strain mice. The WEHI-3 cells are a murine leukemia cell line, specifically derived from the BALB/c strain mice. The WEHI-3 cells were cultured in Iscove's Modified Dulbecco's Medium (IMDM) added with 1% penicillin, 0.05 mM 2-Mercaptoethanol and 10% heat-inactivated fetal bovine serum at 37℃and 5% CO^2^ incubator. This WEHI-3 cells are extensively used as a model to study the immune response, particularly the mechanisms underlying the response of leukemic cells to various treatments. The EL4 mouse thymoma cells are used for a variety of research purposes, including studies on tumor immunology, cell signaling, apoptosis, necrosis and drug response. The origin of EL4 cells from murine thymoma makes them a relevant model for studying T-cell biology, providing insights into T cell development, and T cell function. The AKT plasmid that has constitutively active activity has been previously described 22. A vaccine strain of *Salmonella enterica* serovar* choleraesuis* (S. Choleraesuis; S.C.) was obtained from Bioresources Collection and Research Center (Hsinchu, Taiwan) 21. Animal experiments used C57BL/6 mice purchased from the National Laboratory Animal Center of Taiwan. The Laboratory Animal Care and Use Committee of the National Sun Yat-sen University approved the animal experimental protocol (permit number: 10829).

### Cell viability assay

B16F10 and LL2 cells (5 × 10^4^ cells/well) were plated in 96-well and incubated for one day, then cells were treated with arbutin (0.39, 0.78, 1.56 μM) in serum-free medium for 6 h, cisplatin (2 µg/ml) as positive control. Cell proliferation was evaluated by the WST-1 Cell Proliferation Assay Kit (Sigma-Aldrich) according to the manufacturer's instructions, and the absorbance was measured at the indicated times using the SPECTROstar Nano Microplate Reader. The cells were seeded in 96-well culture plates (10^4^/well), then treated by arbutin (0.39-1.56 µM) for 6 h, cisplatin (16 µg/ml) as positive control. Cell proliferation was examined with the 5-bromo-2'-deoxyuridine (BrdU) Cell Proliferation Fluorescence Imaging Kit (AAT Bioquest, US) uses BrdU which was incorporated into cellular DNA during DNA synthesis. After fixing cells, the incorporated BrdU is labelled with iFluor® 488 MTA. The images were taken with the Olympus fluorescence microscopy 23.

### Flow cytometry

B16F10 and LL2 cells were seeded in 6-well plates at a density of 5 × 10^5^ cells per well and incubated for 24 h. Following this, the cells were treated with either PBS or arbutin at a concentration of 1.56 μM in a serum-free medium for 6 h. For PD-L1 detection, cells were harvested (Use gentle methods to detach cells, such as using cell scrapers instead of enzymatic treatments (e.g., trypsin) which can cleave surface proteins. Include protease inhibitors in the buffers during cell harvesting to protect surface proteins from degradation. Quickly process and fix the cells after harvesting to preserve surface marker expression), counted to ensure 5 × 10^5^ cells, and then fixed in 70% ethanol at 4°C overnight. The fixed cells were then incubated with a PD-L1 primary antibody (Dilution: 1:2000) (GeneTex, Inc. Irvine, CA, USA) for 1 h at 4°C. This was followed by a 30-minute incubation with a fluorochrome-labeled goat anti-rabbit IgG secondary antibody (GeneTex Inc. Irvine, CA, USA) at the same temperature. The samples were subsequently analyzed using the Attune NxT Flow Cytometer (Life Technologies, Carlsbad, CA, USA).

### Western blot analysis

Arbutin influenced protein expression in B16F10 and LL2 cells. B16F10 and LL2 cells (5 × 10^5^ cells/well) were placed into 6-well plates and incubated at 37 °C for 24 h. Then treatment with arbutin (0-1.56 μM) for 6 h, the expression of proteins was measured by Western blotting. To determine protein concentrations, we employed the Bicinchoninic Acid (BCA) Protein Assay kit (Pierce Biotechnology, Rockford, IL, USA). The protein samples underwent separation via SDS-PAGE, after which the resolved proteins were transferred onto a polyvinylidene fluoride (PVDF) membrane (Pall Life Science, Glen Cove, NY, USA). The membrane was then probed with the following primary antibodies: PD-L1 (Dilution: 1:1000) (GeneTex), phospho-AKT (Dilution: 1:1000) (Santa Cruz Biotechnology Inc, Santa Cruz, CA, USA), AKT (Dilution: 1:1000) (Santa Cruz Biotechnology), phospho-mTOR (Dilution: 1:1000) (Cell Signaling, Danvers, MA, USA), mTOR (Dilution: 1:1000) (Cell Signaling), caspase 3 (Dilution: 1:1000) (GeneTex), LC3 (Dilution: 1:1000) (Novus Biologicals, Littleton, CO, USA) and a monoclonal antibody against β-actin (Dilution: 1:10000) (Sigma-Aldrich). We used either horseradish peroxidase-conjugated goat anti-mouse IgG or anti-rabbit IgG (Dilution: 1:20000) (both from Jackson ImmunoResearch Inc, West Grove, PA, USA) for secondary detection. The bound protein-antibody complexes were then visualized using the enhanced chemiluminescence system (T-Pro Biotechnology, New Taipei City, Taiwan). The resulting signals were quantified using the ImageJ software 24.

### Co-culture system

B16F10 and LL2 cells (5 × 10^5^ cells/well) were plated in 6 well-plates and treated with arbutin (1.56 μM) for 6 h. The control cells were treated with PBS. The supernatant was removed, and added the immune cells, murine EL4 lymphoblast cells, or murine WEHI-3 leukemia, which were mixed with an equal amount of serum-free medium. After 24 h, the immune cells were collected and the protein expression for Western blotting was detected and cell survival was assessed using the trypan blue exclusion assay 25.

### Animal study

Six- to eight-week-old C57BL/6 mice were subcutaneously inoculated with 10^6^ B16F10 or LL2 cells at day 0. The mice were intraperitoneally injected with arbutin (50 mg/kg) for 7 consecutive days from day 8 to day 14. The control mice were treated with PBS. Tumor size and body weight were measured every 3 days. The volume of palpable tumors was calculated using the following formula: (length of tumor) × (width of tumor)^2^ × 0.45. Five mice were sacrificed in each group on day 14 and the tumors were taken down for further analysis.

### Statistical analysis

Experimental data were analyzed and reported as mean ± standard deviation (SD).

To evaluate statistically significant differences, the Student's t-test was utilized to evaluate statistically significant differences. The Student's t-test assumes that the data is approximately normally distributed. The primary purpose of the Student's t-test is to compare the means of two groups to determine if they are statistically different from each other. This is particularly useful in experiments where we want to assess the effect of a treatment or intervention compared to a control group. The* p*-value less than 0.05 was considered to be statistically significant.

## Results

### Ideal concentration of arbutin

**Figure 1:**
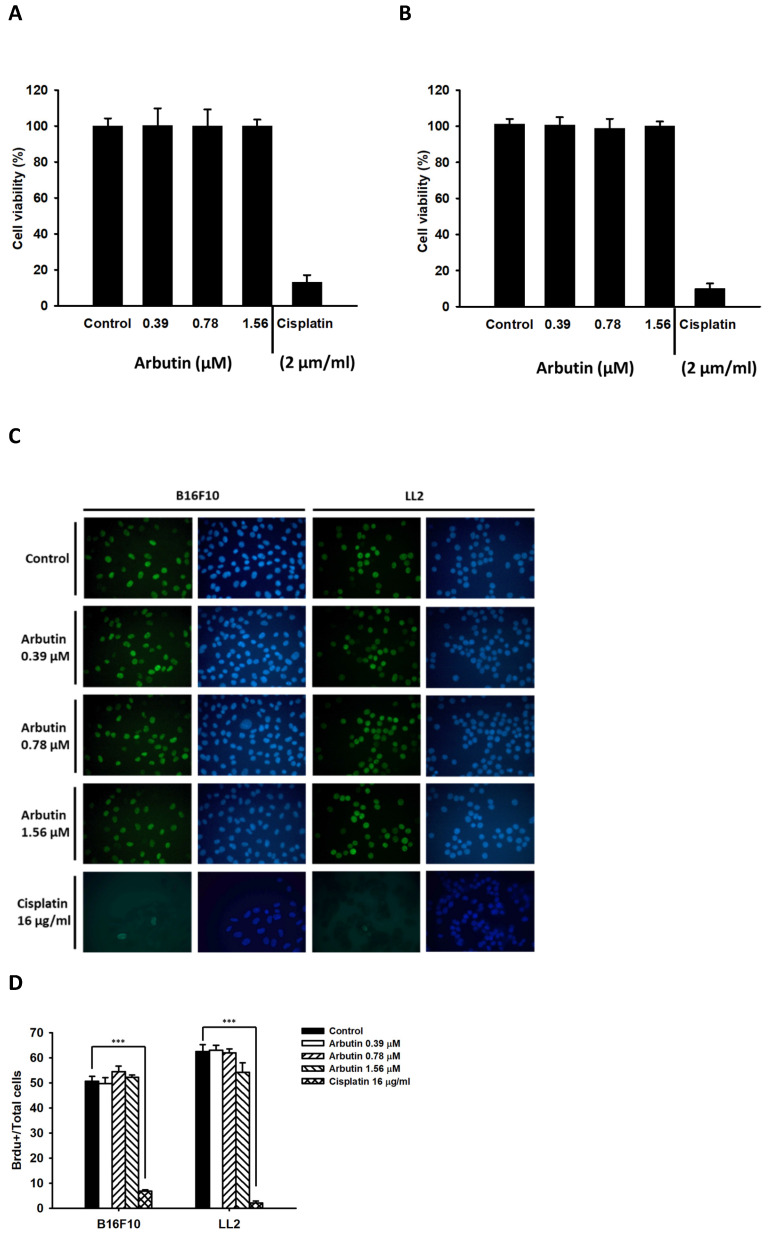
** Effect of Arbutin on cell viability in B16F10 and LL2 cell.** The B16F10 and LL2 cells (5 × 10^5^ cells/well) were placed into 96-well plates and incubated at 37℃ for 24 h. The B16F10 (A) and LL2 (B) cells were treated with indicated concentrations of arbutin or cisplatin (2 µg/ml) for 6 h. Cell viability was evaluated by WST-1 cell proliferation assay. (mean ± SD, n=5). The B16F10 and LL2 cells (5 × 10^4^ cells/well) were placed into 96-well plates and incubated at 37℃ for 24 h. The B16F10 and LL2 cells were treated with indicated concentrations of arbutin for 6 h. (C) Then the cells were fixed and stained for BrdU (green) and nuclei were counterstained with Hoechst 33342 (blue). (D) The cells were counted under a fluorescence microscope (mean ± SD, n=3; ***, *p* < 0.001).

Treatment options for metastatic tumor are severely limited, but recent immunotherapy approaches targeting immune checkpoints have shown great promise in various cancers, including melanoma and non-small cell lung cancer (NSCLC). In this study, we utilized an immunocompetent murine model of melanoma and NSCLC to investigate the effects of arbutin administration on tumor growth. In this investigation, we utilized murine melanoma B16F10 and murine lung carcinoma LL2 cells to examine the anti-tumor potential of arbutin. The cell viability assay, performed on both B16F10 and LL2 cells over 6 h with varying concentrations of arbutin, is shown in Figure 1. Our findings revealed that arbutin did not inhibit cell proliferation in B16F10 (Figure 1A) or LL2 cells (Figure 1B) across the tested doses. Cisplatin induced cell death as positive control (Figure 1 A and B). We hope to find a concentration of arbutin that has immunomodulatory effects without having direct cytotoxic effects on the cells. Previously, arbutin effectively induced apoptosis in the C6 glioma cells and the IC50 dose was obtained at 30 µM 26. To monitor cell proliferation influence by arbutin in B16F10 and LL2 cells, we use BrdU fluorescence to investigate the range of 0-1.56 µM arbutin (Figure 1C). These cells treated with cisplatin as positive control. Figure 1D showed that arbutin did not significantly inhibited cell proliferation in these cells in a dose-dependent manner by quantification of BrdU-positive cells. Based on these observations, these dosages were subsequently chosen to assess the influence of arbutin on PD-L1 expression.

### *In vitro* influence of arbutin on PD-L1 expression

**Figure 2:**
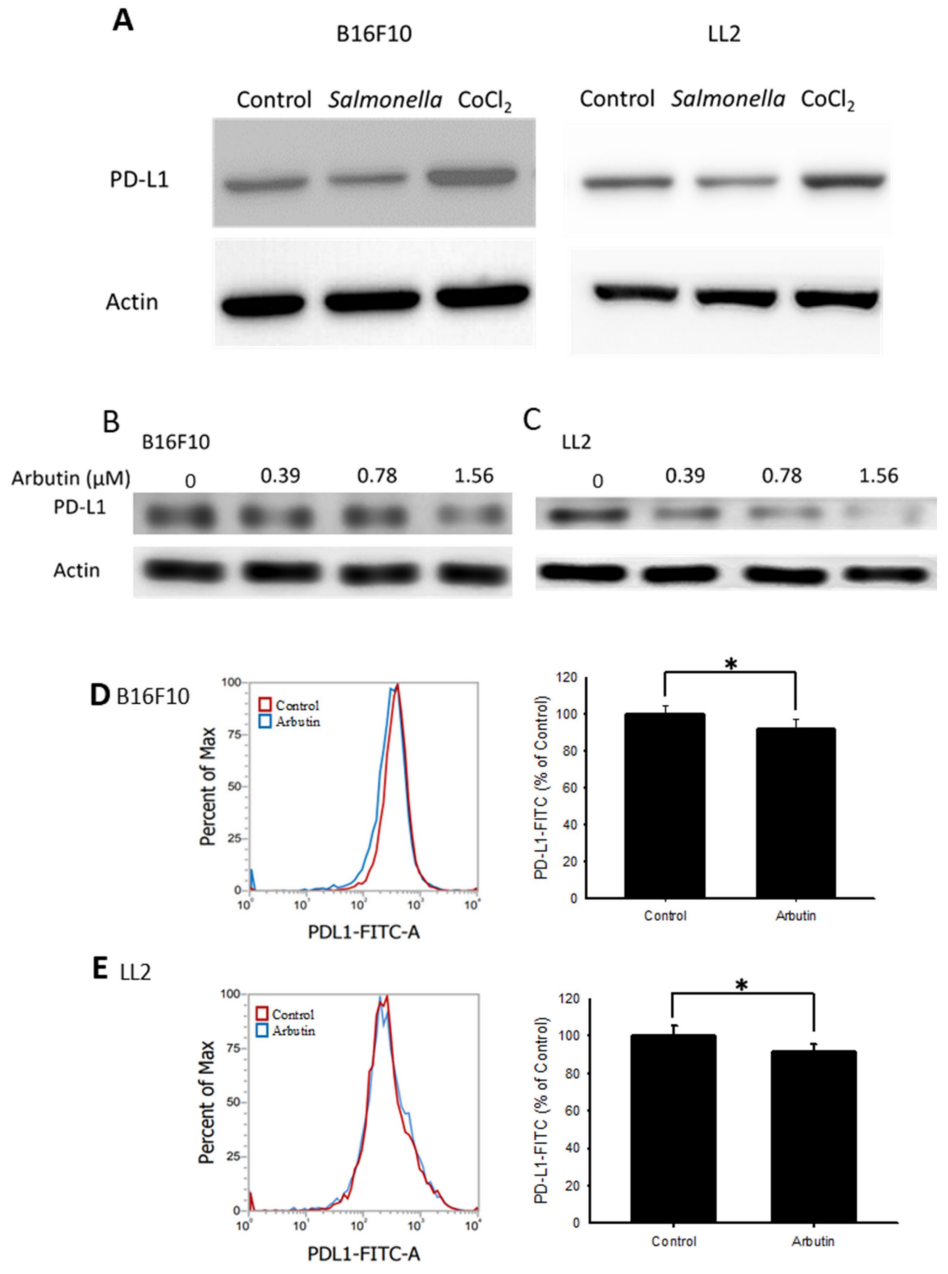
** Arbutin inhibited PD-L1 expression in B16F10 and LL2 cells.** Arbutin reduced PD-L1 expression in B16F10 and LL2 cells. (A) B16F10 and LL2 cells (5 × 10^5^ cells/well) were placed into 6-well plates and incubated at 37 °C for 24 h. Then treatment with* Salmonella* (5 × 10^7^ cells/well) for 1.5h or CoCl_2_ (200 μM) for 6 h, the expression of PD-L1 was measured by Western blotting. B16F10 (B) and LL2 (C) cells (5 × 10^5^ cells/well) were placed into 6-well plates and incubated at 37 °C for 24 h. Then treatment with arbutin (0-1.56 μM) for 6 h, the expression of PD-L1 was measured by Western blotting. Arbutin reduced PD-L1 expression on the surface of cells. After treatment with arbutin (1.56 μM) for 6 h, the expression of PD-L1 on the surface of B16F10 (C) and LL2 (D) cells was measured by flow cytometry. (mean ± SD, n=5; *, *p* < 0.05).

The role of the PD-L1-mediated immune checkpoint within the tumor microenvironment is critical for tumor immune evasion 27. We examined the protein levels of PD-L1 in both B16F10 and LL2 cells. Previous studies indicated that *Salmonella* inhibit PD-L1 expression 13 and hypoxia (CoCl_2_ simulates anaerobic environment) enhanced PD-L1 expression 28. *Salmonella* reduced PD-L1 expression and CoCl_2_ increased PD-L1 expression (Figure 2 A). Figure 2 B and C shows the PD-L1 levels in tumor cells following various arbutin treatments over 6 hr. Compared to the control, there was a noticeable decline in PD-L1 expression in both B16F10 (Figure 2 B) and LL2 cells (Figure 2 C) with incremental arbutin exposure. Flow cytometry analysis further revealed a significant reduction in cell surface PD-L1 expression post-treatment with 1.56 μM of arbutin in B16F10 (Figure 2 D) and LL2 cells (Figure 2 E). These experimental conditions do not trigger autophagy (Figure S1). These findings indicate that arbutin can dose-dependently suppress the surface expression of PD-L1 on tumor cells, without affecting their cell viability.

### Arbutin modulates PD-L1 expression in tumor cells via the AKT/mTOR signaling pathway

**Figure 3:**
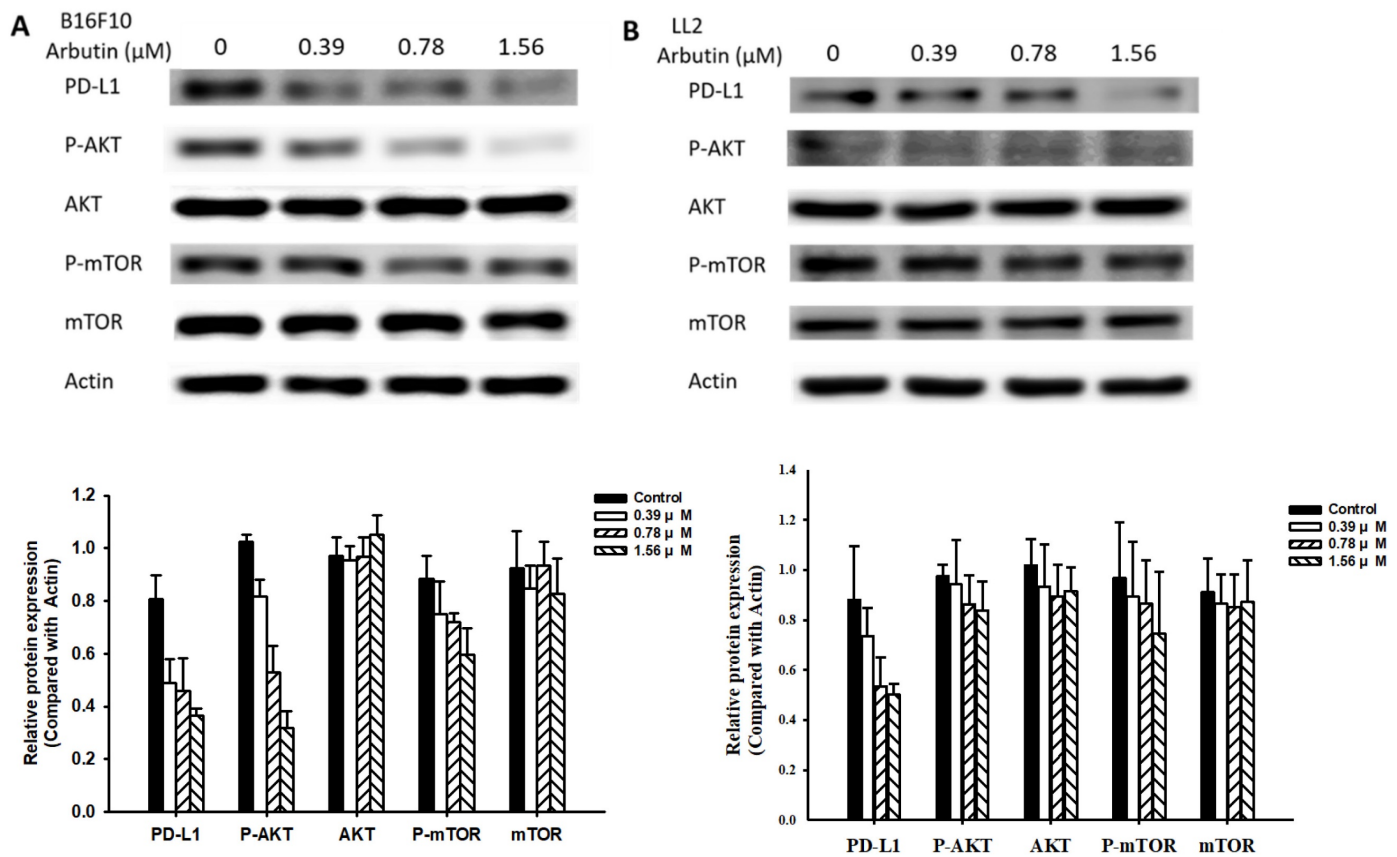
** Arbutin-mediated PD-L1 protein expression.** B16F10 (A) and LL2 (B) cells (5 × 10^5^ cells/well) were placed into 6-well plates and incubated at 37 °C for 24 h. Then treatment with arbutin (0-1.56 μM) for 6 h, the expression of PD-L1 and phosphorylation-AKT/mTOR were measured by Western blotting. Quantification histograms are presented beneath each Western blotting plot. Data are expressed as the mean ± SD of three-time repeated determinations. Each experiment was repeated three times with similar results.

**Figure 4:**
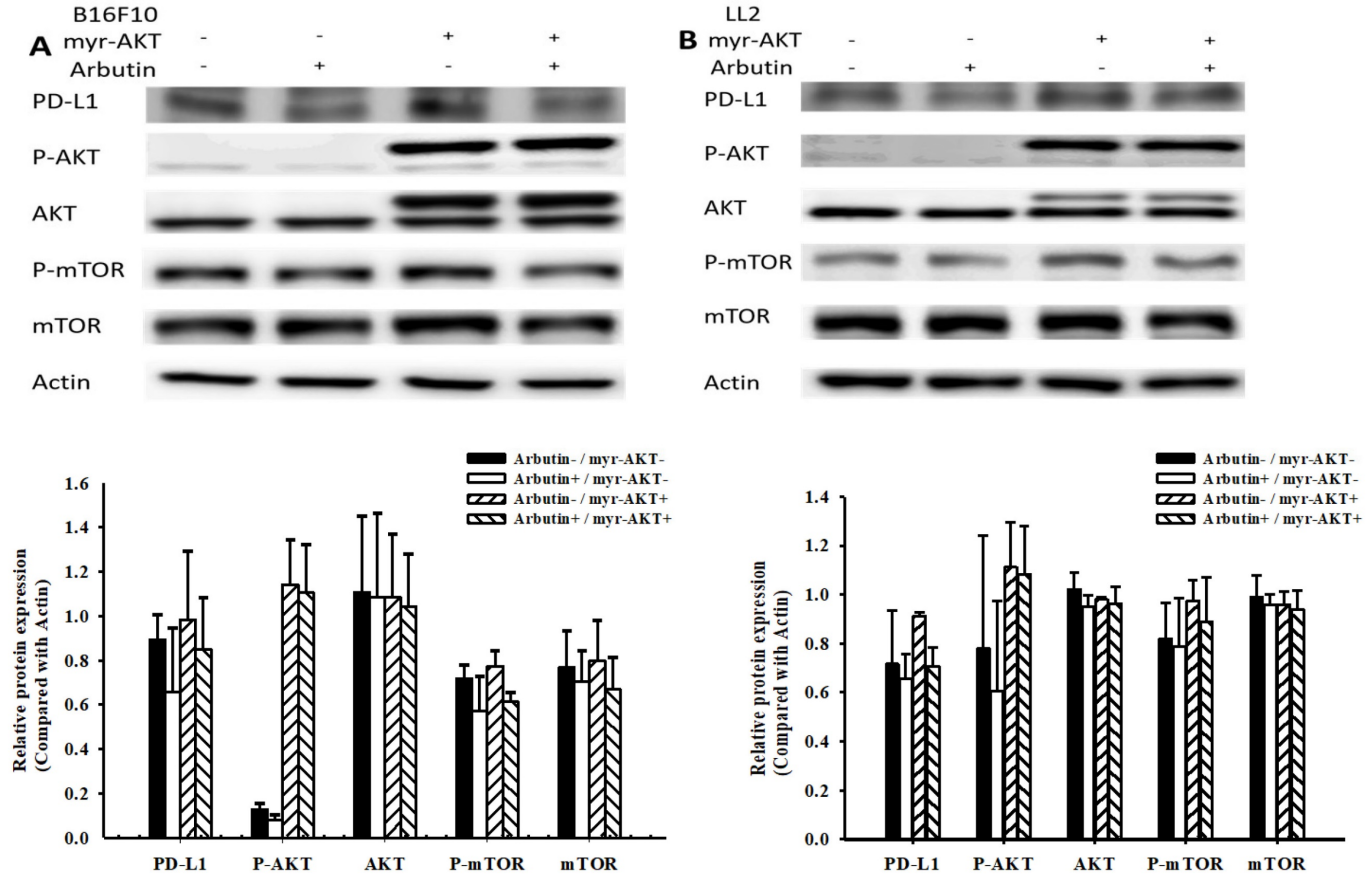
** Arbutin reduces PD-L1 expression through the AKT/mTOR pathway.** B16F10 (A) and LL2 (B) cells (5 × 10^5^ cells/well) were placed into 6-well plates and incubated at 37 °C for 24 h. Transfect the cells with control or constitutively active AKT plasmid (5ug) at 37°C for 6 h, then treat with arbutin (1.56 μM) for 6 h. Analyze the expression levels of the AKT/mTOR proteins and PD-L1 in the cells using Western blotting. Quantification histograms are presented beneath each Western blotting plot. Data are expressed as the mean ± SD of three-time repeated determinations. Each experiment was repeated three times with similar results.

In this study, we delved deeper into the mechanistic influence of arbutin on tumors. Activation of the PI3K/AKT signaling pathway plays a pivotal role in tumor cell survival, proliferation, and motility. Past studies have posited that PD-L1 expression is governed by the AKT/mTOR signaling pathway 13. Moreover, the activation of this signaling pathway has been associated with enhanced PD-L1 expression. With this background, we evaluated the expression levels of PD-L1, phosphorylated AKT, and phosphorylated mTOR, as shown in Figure 3. Arbutin treatment diminished the phosphorylation levels of AKT and mTOR, pointing towards its inhibitory effect on the AKT/mTOR pathway in B16F10 (Figure 3A) and LL2 cells (Figure 3B). To further substantiate the role of arbutin in modulating PD-L1 expression through AKT phosphorylation, we employed a constitutively active AKT plasmid to restore AKT/mTOR signaling. Figure 4 indicates that post-transfection with the active AKT plasmid, there was a noticeable elevation in PD-L1 expression, accompanied by an increase in AKT/mTOR phosphorylation. Relative to the control transfection, the active AKT plasmid-transfected cells showed augmented PD-L1 expression following arbutin exposure, observed in B16F10 (Figure 4A) and LL2 cells (Figure 4B). Our results underscore the importance of arbutin's downregulation of AKT phosphorylation in diminishing PD-L1 expression in B16F10 and LL2 cells. Arbutin essentially hinders tumor PD-L1 expression by orchestrating changes in the AKT/mTOR signaling pathway.

### Impact of arbutin treatment on immune cell protein expression

**Figure 5:**
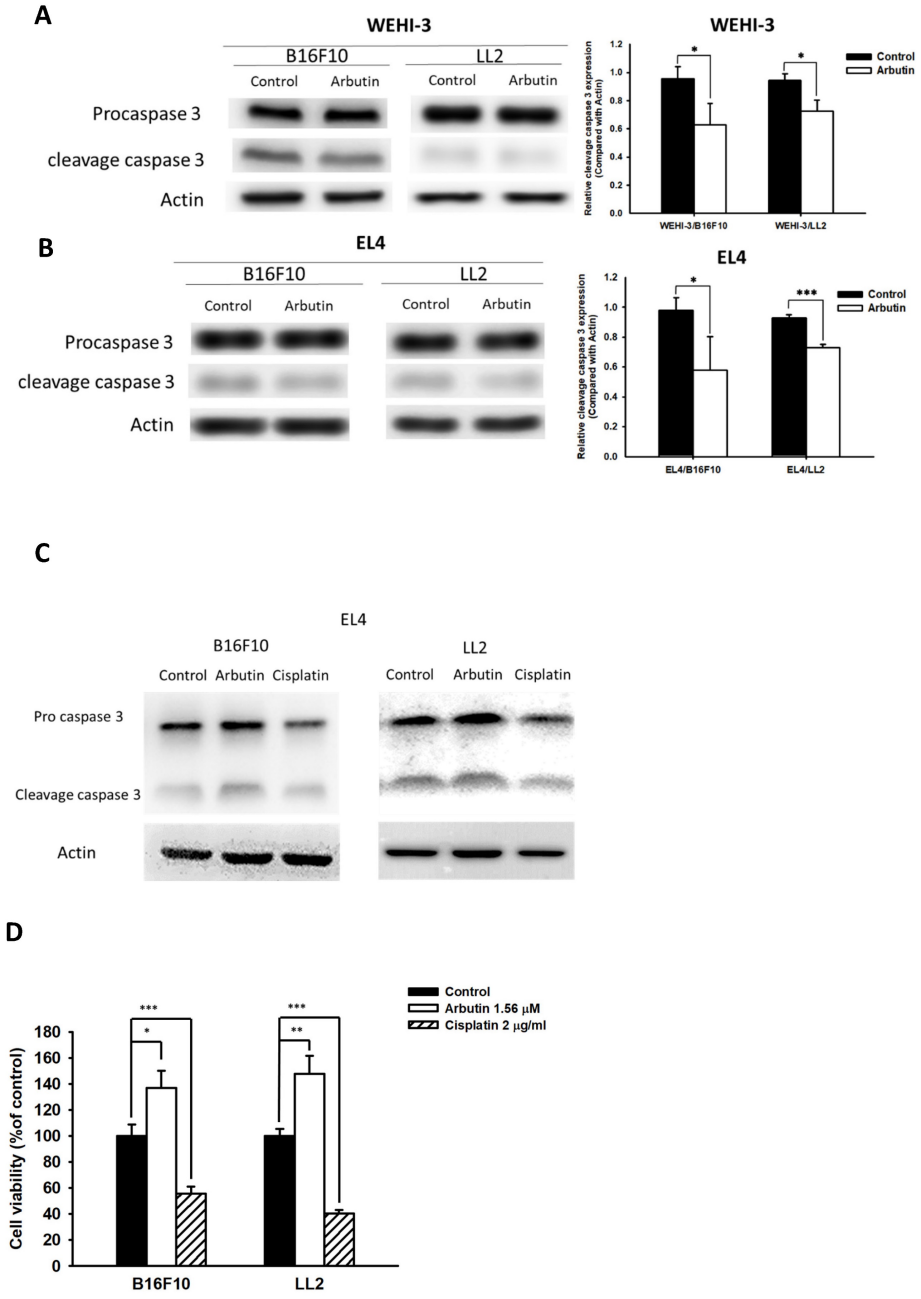
** Arbutin affected apoptosis in immune cells.** B16F10 and LL2 cells (5 × 10^5^ cells/well) were placed into 6-well plates, incubated at 37 °C for 24 h, and then treated with arbutin (1.56 μM) for 6 h. WEHI-3 (A) and EL4 (B) cells were co-cultured with arbutin-treated B16F10 and LL2 cells before harvesting. The protein expression was analyzed by Western blotting. (C) EL4 cells were co-cultured with arbutin-treated B16F10 and LL2 cells or treated with cisplatin (2 µg/ml) before harvesting. The protein expression was analyzed by Western blotting. (D) The EL4 cell number were measured by staining with trypan blue. (n = 6, data are mean± SD; * *p* < 0.05; **, *p* < 0.01; *** , *p* < 0.001). Each experiment was repeated three times with similar results.

Previous research has illuminated that PD-L1 is abundantly expressed in human lung cancer and melanoma, with high levels of PD-L1 in tumors potentially triggering T-cell apoptosis 13. In light of this, we sought to discern whether the observed decrease in PD-L1 after co-culturing immune cells with tumor cells played a pivotal role in the effects elicited by arbutin on immune cell activity. For this, we chose mouse EL4 lymphocytes and WEHI-3 leukemia cells to co-culture with B16F10 and LL2 tumor cells treated with 1.56 μM arbutin. Results from the Western blot analysis highlighted a marked decrease in cleaved caspase 3 in WEHI-3 (Figure 5 A) and EL4 (Figure 5 B and C) cells when co-cultured with arbutin-treated tumor cells. In Figure 5 C, cisplatin induced cell apoptosis as positive control. Furthermore, the EL4 cell viability was higher after the co-cultured with arbutin-treated tumor cells than that derived from the tumor cells treated with PBS (Figure 5 D). These findings attest to the inhibitory effect of arbutin on the production and functionality of PD-L1 in tumor cells.

### Arbutin suppresses PD-L1 expression and tumor growth* in vivo*

**Figure 6:**
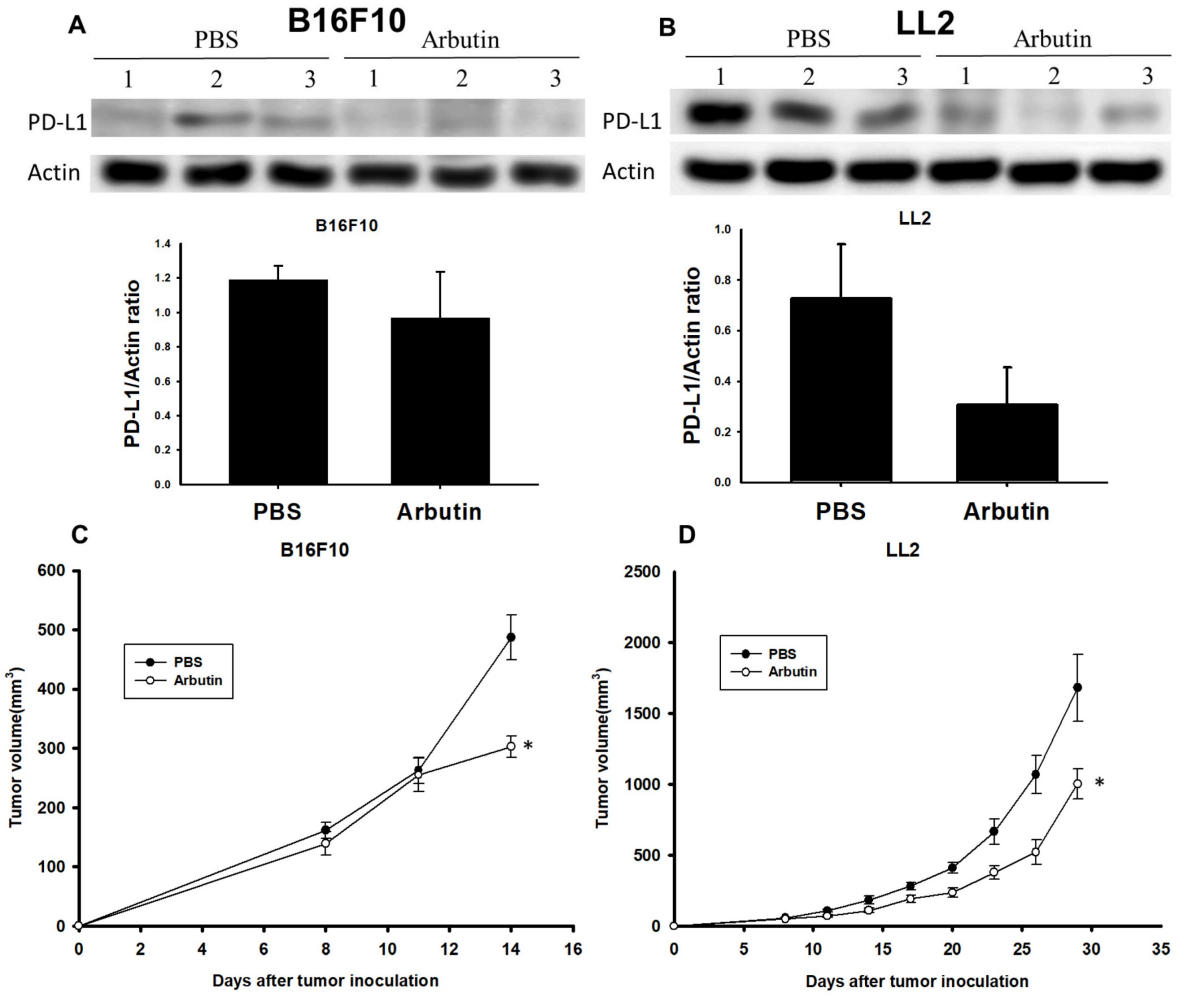
** Arbutin inhibits tumor growth and PD-L1 expression *in vivo*.** B16F10 and LL2 tumor cells were subcutaneously injected into C57BL/6 mice on day 0. Tumor growth was allowed for seven days, and arbutin (50 mg/kg) was administered via intraperitoneal injection for seven consecutive days from day 8. Tumor volumes were measured every three days. On Day 15, tumors were extracted using a lysis buffer. The supernatant was collected, and PD-L1 expression levels were analyzed using Western blotting for (A) B16F10 (n=3) and (B) LL2 (n=3) tumors. Tumor volumes were compared between the control group and the arbutin-treated group for (C) B16F10 (n=10) and (D) LL2 (n=9) tumors. (mean ± SEM; *, *p* < 0.05).

To validate the inhibitory effect of arbutin on tumor growth in a live model, C57BL/6 mice were subcutaneously injected with either B16F10 or LL2 cells (10^6^) on day 0. Starting on day 7, the mice received intraperitoneal injections of arbutin (50 mg/kg) or PBS daily for consecutive seven days. After this treatment duration, tumors were harvested, processed, and analyzed for PD-L1 expression levels. Figure 6A reveals that PD-L1 expression was notably suppressed in the arbutin-treated B16F10-bearing mice compared to the PBS control group. A similar trend was observed in LL2-bearing mice, as illustrated in Figure 6B. Furthermore, the tumor volume was monitored every three days starting from day 7. The data demonstrated that the arbutin-treated group experienced a significant reduction in tumor growth volume compared to the PBS control group in both tumor models (Figure 6 C and D). This study conclusively showed that murine melanoma and lung tumor cells exhibited markedly decreased growth following arbutin treatment.

## Discussion

Immune checkpoint inhibitors are immunotherapies that block immune checkpoints, thereby promoting anti-tumor immune responses. Prominent among these are the CTLA-4 and PD-1/PD-L1 pathways. By inhibiting checkpoint proteins, these therapies can rejuvenate T-cell activity and contribute to tumor regression 27. However, expanding the use of immune checkpoint inhibitors in clinical settings has exposed significant challenges, including the risk of collateral effects on the immune system 29. Arbutin stands out due to its range of properties: it has a whitening effect, is anti-inflammatory, and possesses antioxidant properties that impede melanin formation. Prior research has highlighted its capability to down-regulate proteins related to tumor growth 30. Our investigations indicate that arbutin exerts its effects primarily through immunomodulation, rather than direct cytotoxicity to tumor cells (Figure 1 and 2). Its treatment reduces PD-L1 expression at both surface and protein levels, underscoring its potential as an immunotherapeutic agent. Arbutin bolsters host immunity by attenuating effector T cell apoptosis through decreased PD-L1 expression.

Arbutin indeed reduced the expression of PD-L1 in this study. The reduction in surface PD-L1 by arbutin (Figure 2 D and E) was not as significant as the reduction observed in total PD-L1 (Figure 2 B and C). Arbutin may indeed reduce the intrinsic PD-L1 levels in tumor cells, thereby affecting tumor malignancy. Because arbutin reduces a small amount of surface PD-L1 but significantly inhibits the expression of intrinsic PD-L1, it is expected to have a synergistic effect when combined with anti- CTLA-4 therapy. These findings support the notion that reducing intrinsic PD-L1 expression can synergize with immune checkpoint blockade (ICB) therapy, potentially leading to better clinical outcomes in cancer patients 29.

Though *in vivo* studies have substantiated arbutin's tumor-inhibiting capabilities, additional evidence remains crucial. Notably, PD-L1 in tumor cells promotes immune suppression by increasing IL-10 production in peripheral regulatory T cells 30. The PD-1/PD-L1 interaction inhibits tumor-infiltrating CD4^+^/CD8^+^ T cells, reducing cytokine secretion and facilitating tumor immune evasion 13. Hence, a deeper investigation into arbutin's impact on T cell function is warranted 31-32. Unlike many inhibitors, distinct advantage of arbutin lies in its widespread presence in various plants, allowing easy acquisition through diverse dietary sources. More significantly, its consumption poses no harm to humans. Arbutin has been shown to suppress the proliferation of certain cancer cells by downregulating the AKT/mTOR signaling pathway. This inhibition can lead to reduced tumor cell growth and reduction of inflammation 26. In this study, arbutin may influence the intrinsic functions of PD-L1 by modulating the AKT/mTOR signaling pathway, potentially leading to decreased T/tumor cell proliferation and enhanced apoptosis. This suggests a promising avenue for cancer therapy research, combining arbutin's anti-cancer properties with its effects on crucial signaling pathways involved in tumor progression.

Arbutin, a natural hydroxyquinone glucoside, exists in two configurations: α and β-quercetin glycosides. Current findings highlight that α-glycosides exhibit more significant activity than their β counterparts, leading to α-arbutin's heightened efficacy against tyrosinase. The inhibitory effect of α-arbutin on tyrosinase surpasses that of β-arbutin tenfold 33. This study, however, utilized β-arbutin. A deeper exploration into α-arbutin's effects on tumor cells might offer insights beneficial for drug development or adjuvant therapies. By comprehensively understanding the mechanisms of natural compounds, there's potential for harnessing their anticancer properties, paving the way for innovative cancer treatments.

## Supplementary Material

Supplementary figure.
