# Supplementary material for: Arbutin overcomes tumor immune tolerance by inhibiting tumor programmed cell death-ligand 1 expression
Source: Int J Med Sci. 2024 Nov 11;21(15):2992–3002. doi: 10.7150/ijms.92419 (PMC11610324; doi:10.7150/ijms.92419)
Supplement: Supplementary file 1 — Supplementary figure. [file ijmsv21p2992s1.pdf]

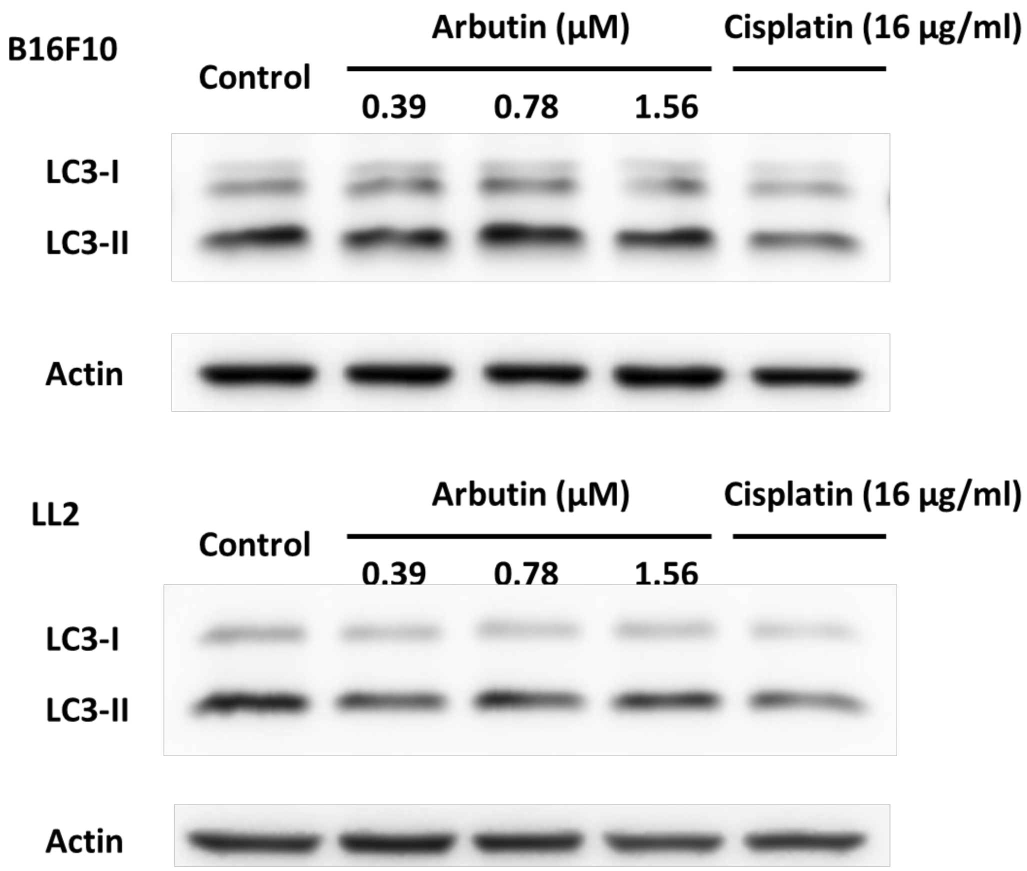

Figure S1. Arbutin did not induce autophagy in B16F10 and LL2 cells. Arbutin reduced PD-L1 expression in B16F10 and LL2 cells. B16F10 (A) and LL2 (B) cells (5×10^5 cells/well) were placed into 6-well plates and incubated at 37 °C for 24 h. Then treatment with arbutin (0-1.56 μM) for 6 h, the expression of LC3-I and LC3-II was measured by Western blotting, cisplatin as positive control.
